# Recontacting patients in clinical genetics services: recommendations of the European Society of Human Genetics

**DOI:** 10.1038/s41431-018-0285-1

**Published:** 2018-10-11

**Authors:** Daniele Carrieri, Heidi C. Howard, Caroline Benjamin, Angus J. Clarke, Sandi Dheensa, Shane Doheny, Naomi Hawkins, Tanya F. Halbersma-Konings, Leigh Jackson, Hülya Kayserili, Susan E. Kelly, Anneke M. Lucassen, Álvaro Mendes, Emmanuelle Rial-Sebbag, Vigdís Stefánsdóttir, Peter D. Turnpenny, Carla G. van El, Irene M. van Langen, Martina C. Cornel, Francesca Forzano, On behalf of the European Society of Human Genetics

**Affiliations:** 1 University of Exeter, Egenis, UK; 2 Centre for Research Ethics and Bioethics, Uppsala University, Uppsala, Sweden; 3 School of Community Health & Midwifery, University of Central Lancashire (UCLan), Preston, England UK; 4 Merseyside and Cheshire Clinical Genetics Service, Liverpool Women’s NHS Hospital Trust, Liverpool, England UK; 5 School of Medicine, Cardiff University, United Kingdom 9 School of Medicine, University of Exeter, Egenis, UK; 6 Clinical Ethics and Law, Faculty of Medicine, University of Southampton, Southampton, UK; 7 Law School, University of Exeter, Exeter, UK; 8 University of Groningen, University Medical Center Groningen, Department of Genetics, Groningen, The Netherlands; 9 School of Medicine, University of Exeter, Exeter, UK; 10 Medical Genetics Department, Koç University School of Medicine (KUSoM), İstanbul, Turkey; 11 Wessex Clinical Genetics Service, University Hospitals Southampton NHS Foundation Trust, Southampton, UK; 12 UnIGENe and CGPP – Centre for Predictive and Preventive Genetics, IBMC – Institute for Molecular and Cell Biology, i3S – Instituto de Investigação e Inovação em Saúde, Universidade do Porto, Porto, Portugal; 13 UMR 1027, Inserm, Université Paul Sabatier-Toulouse III, Toulouse, Cedex France; 14 Landspitali – the National University Hospital of Iceland, Department of Genetics and Molecular Medicine, Reykjavík, Iceland; 15 Clinical Genetics, Royal Devon & Exeter NHS Foundation Trust, Exeter, UK; 16 Section Community Genetics, Department of Clinical Genetics and Amsterdam Public Health research institute, Amsterdam UMC, Vrije Universiteit, Amsterdam, The Netherlands; 17 Clinical Genetics Department, Guy’s & St. Thomas’ NHS Foundation Trust, London, UK

**Keywords:** Health services, Genetic counselling

## Abstract

Technological advances have increased the availability of genomic data in research and the clinic. If, over time, interpretation of the significance of the data changes, or new information becomes available, the question arises as to whether recontacting the patient and/or family is indicated. The Public and Professional Policy Committee of the European Society of Human Genetics (ESHG), together with research groups from the UK and the Netherlands, developed recommendations on recontacting which, after public consultation, have been endorsed by ESHG Board. In clinical genetics, recontacting for updating patients with new, clinically significant information related to their diagnosis or previous genetic testing may be justifiable and, where possible, desirable. Consensus about the type of information that should trigger recontacting converges around its clinical and personal utility. The organization of recontacting procedures and policies in current health care systems is challenging. It should be sustainable, commensurate with previously obtained consent, and a shared responsibility between healthcare providers, laboratories, patients, and other stakeholders. Optimal use of the limited clinical resources currently available is needed. Allocation of dedicated resources for recontacting should be considered. Finally, there is a need for more evidence, including economic and utility of information for people, to inform which strategies provide the most cost-effective use of healthcare resources for recontacting.

## Introduction

### Setting the scene

Advances in genomic technologies have led to a reduction in both the cost and speed of genome sequencing, generating unprecedented amounts of data. Genomic data may provide information about predisposition to, and/or diagnosis and treatment of, many health conditions, contributing to improved preventative therapies or surveillance regimens, as well as targeting treatments to sub-groups of patients [1]. Amalgamation of genomic data from different populations, acquired via health care or research purposes, and their correlation with phenotypic data further adds to understanding the genome by analyzing data on frequency and pathogenicity of variants, better grouping of phenotypes, and information on the natural history of various conditions.

A positive outcome of this increase in data collection is the generation of more knowledge about the effect or pathogenicity of genomic variants previously classified as ‘variants of uncertain significance’ (VUSs; ‘variant of unknown significance’ and ‘variant of uncertain significance’ in this document are used as synonymous) [2], and potentially to revising their first interpretation. However, it also raises many questions about whether, and how, to store and use this genomic data in the future. For example, if a patient has had many genes sequenced, or has had their whole exome or genome sequenced, should the genomic data (raw or interpreted) be stored and referred to in the future, as new information about variant-trait associations is discovered? And if so, who would or should have the responsibility to re-evaluate the genomic data? Who, therefore, should ‘recontact’ the patient and family if there are new implications of clinical importance? Should this be the patient’s clinical care team or the clinical genetics service (who may have had no contact with the patient)? Should the genetic diagnostics laboratory initiate this process, in the case of updates of previous genetic reports? What role do patients play? Would exome or genome reinterpretation, and (potentially) recontacting, be considered for all patients or would prioritization be necessary, e.g., for those who did not receive a molecular diagnosis when the original sequencing was performed? Would information on variants related to the initial clinical problem be prioritized? What would the logistics of the process of recontacting look like, and what are the resource implications? Examples of recontacting scenarios are given in Box 1.

The ESHG recommendations on whole-genome sequencing emphasize the need for more professional guidance on recontact practices [3]. It is also important to highlight that the question of *when* to recontact is further complicated by the complexity, and often the uncertainty, of genomic information [4], meaning that recontact triggers can be difficult to define [5]. Furthermore, what constitutes sufficient evidence of pathogenicity/effect, or the lack thereof, to justify the need to trigger recontact? [1]. As more evidence is gathered, some uncertainty is likely to resolve over time, which will bring the issue of recontacting into focus. These issues become even more problematic [6] as genetic testing, including whole exome and whole genome sequencing, is increasingly used in mainstream medical practice, e.g., cardiology or oncology [7]. At the core of these questions is the tension between recognising that appropriate recontacting could be crucial to maximizing the benefits of new genomic technologies, by improving medical and/or psychosocial care for patients and/or their family members, and identifying the resources that will enable these benefits to be realized while also minimizing potential harm.

Box 1 Examples of scenarios for recontacting and the questions raised CLINICAL GENETIC HEALTH CARE SCENARIO

A child with developmental delay was referred to clinical genetics in 2004 as the older sister was pregnant and expressed concern regarding recurrence risk in the family. Unfortunately, no clinical diagnosis was made. A variant in an X-linked gene was detected, but its significance was uncertain. The sister delivered two healthy daughters. A decade later the significance of the variant is modified: it is now considered a pathogenic variant. How should this information reach the family? Should the lab contact the clinical geneticist? Should the clinical geneticist recontact the family? Should the lab send a new report to the family? Should the lab send the report to the primary HCP caring for this child?

RESEARCH SCENARIO

A child with intellectual disability was referred for clinical genetics consultation in 2005. Unfortunately, no diagnosis was made. The parents agreed that the information on the phenotype and the DNA could be used for scientific research but the consent form did not address the issue of recontacting. Ten years later exome sequencing became available as a diagnostic tool. In a research project on intellectual disability the child’s DNA was reanalysed and a likely diagnosis found which might change the medical treatment of this child. Should the parents be recontacted to inform them of the research findings? If so, who should initiate the recontact—the researcher or clinician in charge of the family?

NEW GENE DISCOVERY SCENARIO

A new disease gene is discovered, explaining the cause of a complex congenital heart defect in a group of previously undiagnosed patients, with relevant implications for their families. Many Clinical Genetics Centres might have seen such patients over the years. While all clinical teams bear this new diagnosis in mind with any new referral, what should happen to the patients they have seen in the past who are likely to have this new diagnosis?

Recontacting their previous patients and informing them about the possibility of genetic testing therefore arises. To date this has largely happened on an ad hoc basis but what can be done to make this more equitable, considering the scarcity of resources and infrastructure limitations?

### Guidelines and consensus

A statement by the American College of Medical Genetics (ACMG) [8], published in 1999 (currently under revision, personal communication), is so far the only set of guidelines in English that specifically addresses recontacting in clinical practice. It identified the primary care physician as the key healthcare professional (HCP) responsible for alerting patients about the potential need to recontact, since they provided ongoing care and were therefore best placed to initiate and coordinate recontact. The primary care physician was expected to ask the patient about any new developments in the family history and to alert the patient to new potential developments that could affect their health and, if needed, to act as gatekeeper between the patient and secondary and tertiary care services, including clinical genetics Box 2.

Ten years after the ACMG guideline, in light of the increased uptake of genome analysis in the healthcare setting, Shirts and Parker [9], among others, recognized that diagnostic genetics laboratories (where sequencing occurs) may be suitably placed to know about new information that may change the previous interpretation of a variant. Therefore, they suggest not only that laboratories should make an effort to update relevant HCPs accordingly, but also that they might have a responsibility to directly inform clients of changes in test interpretation.

Later, in 2015, EuroGentest and the European Society of Human Genetics (ESHG) issued Guidelines for diagnostic next-generation sequencing, which emphasize that, if reinterpretation of variants occurs over time, the laboratory should share some responsibility in triggering patient recontact [10].

The concept of multidisciplinary collaboration between genetic diagnostics laboratories and genetic HCPs is also in line with ACMG standards and guidelines for the interpretation of sequence variants. Clinical laboratories are encouraged to establish collaborations with clinicians to help improve genotype–phenotype relationships, and to resolve differences in the interpretation of variants between different laboratories [2]. Taken together, these statements identify three important stakeholders in the process of recontacting: HCPs (genetic HCPs and other mainstream specialties, including primary care physicians); patients/families; and scientists from genetics diagnostics laboratories.

The paucity of guidelines reflects the variegated regulatory landscape and a lack of professional consensus around recontacting in clinical practice [6, 11]. Importantly, the latter includes uncertainty about whether recontacting constitutes a duty or responsibility, and if so with whom this would lie, and what criteria should be applied to the newly established information in order to trigger the recontacting process. Indeed, currently, genetic services are struggling with these questions about duties or responsibilities, and how these might be executed [6]. Unsurprisingly, a survey of regulation and practices of genetic counselling in 38 European countries found that recontacting was not covered well either in national law or applied practice guidelines [12]. In ethical terms, genetic HCPs increasingly feel a *moral* duty to recontact, even in the absence of a *legal* duty to do so. Whether or not legal issues and practical issues (resources permitting) allow for recontact may differ among countries [12]. For instance, in Norway a very restrictive data protection regulation is in place, so that recontacting patients is not allowed and new contacts can only be engendered through a new referral or if the patients themselves contact the service.

Box 2 Definitions *Recontact*: Establish a new contact—initiated by either former patient or clinician – with former patients, seen in the past, discharged from care, and no longer in an ongoing relationship with the specific healthcare professional involved.

*Follow-up*: A new appointment, face-to-face or virtual, with a current patient enrolled in ongoing care at the same Clinic/Practice.

*Re-testing*: Conducting a new genetic test on a patient’s sample with an updated or a new technique with the aim to obtain new genetic data.

*Re-analysis*: Using a patient’s existing raw data, that has been sequenced in the past, in order to analyze all genes currently proven to be associated with the patient’s condition (including genes that were not analyzed previously, as a connection with the patient’s condition was not known at the time).

*Re-interpretation*: Re-evaluation of genetic variants that have been analyzed and interpreted in the past, in the light of new available information. This can lead to reclassification and possibly followed by the provision of an updated and modified report of the significance of the data in question.

### Towards ESHG recommendations

As indicated, there is currently a lack of consensus on many issues related to recontacting in clinical practice, but a growing recognition that these issues merit discussion as a matter of priority if genomic approaches to health care are to be realized to their potential, both clinically cost-effectively. Attaining a professional consensus on recontacting is desirable. In this paper, we provide a background to the debate about recontacting in clinical (genetics) practice, as well as Recommendations on Recontacting. A group of authors involved in recent research projects on recontacting in Europe [11, 13–17] (AML, AJC, DC, IMvL, LJ, NH, PDT, SDh, SDo, SEK, TFHK), together with the Public and Professional Policy Committee (PPPC) of the ESHG, elaborated a draft background document integrated with recommendations. These were available online on the ESHG website for membership consultation from 28 March to 30 April 2018, and the members as well as some experts were invited by email to comment. All comments were considered and adaptations made when appropriate. The final, amended draft has been endorsed by the Board of the ESHG on the 15th June 2018.

For the background, we considered: (i) definitions and conceptual clarification of recontacting in clinical practice; (ii) current knowledge and evidence on the issue of recontacting in clinical practice, particularly focused on European countries; (iii) the available ethical and legal opinions and guidance. We focused on recontacting in a clinical, diagnostic setting and have not directly addressed the issues that arise principally in the research setting, though we recognize there is overlap between the two domains.

## Definitions and conceptual clarification

### ‘Follow up’ versus ‘recontact’

An important distinction is that between the notion of ‘recontact’ and that of ‘follow-up’. A systematic review conducted by Otten et al. [6] defines the duty to recontact as the ethical and/or legal obligation to recontact former patients in light of new genetic findings. The term ‘former patients’ allows recontacting to be distinguished from the situation where a current patient receives routine follow-up care. Recontacting then implies updating patients seen in the past, and discharged from care, who are no longer in an ongoing relationship with HCPs, but whose clinical information and genetic data are still accessible to them. Accordingly, recontacting involves identifying and then re-establishing contact with former patients who could benefit from new information related to their health condition.

While this distinction between the follow-up of *current* patients and the recontact of *former* patients is helpful, too much focus on the semantic distinction around these terms might lead us to miss the core of the issue. The difficulty associated with this overly formal approach is the lack of consistency as to whether a patient is (considered) discharged, with variation between specialties, and between different geographical areas even within a single country. There are many drivers for these decisions, such as the funding model for genetic health services in a region or state and other political, legal, economic, historical or practical factors [13]. Some models of health care delivery may result in organisational barriers that could hinder the process of recontacting, such that an action that would be ‘recontacting’ in one genetics service might be regarded as ‘routine clinical follow-up’ in another, and would therefore fall out with the systematic review’s definition of recontacting. Furthermore, most healthcare services are financially stretched and experience intense pressure to work within limited resources which must be distributed in cost-effective ways. In this context, recontacting ‘former’ patients and long-term follow-up both compete with new patient referrals, which are prioritized in many healthcare systems.

Although this argument is largely focused on clinical/medical services, it should be restated that Genetic Laboratories can also be directly involved in recontacting, both directly and indirectly (through HCPs).

### Duty/responsibility

The terms ‘duty’ and ‘responsibility’ to recontact are often used interchangeably in the literature. The word ‘duty’ has gained traction in the recontacting literature from North American papers focusing on legal aspects [6], and conceptualized recontacting as an extension of the legal duty of care [18]. These papers tended to adopt the approach of ‘defensive’ medicine. They focused on the potential medico-legal consequences of HCPs failing to recontact when attempted recontact would be the expected standard of care [19]. Thus, the aim is to limit any potential legal liability, even in the absence of ‘an active physician-patient relationship’ [20]. For clarity, in this paper, we wish to distinguish between a ‘legal duty’, and a ‘professional/ethical responsibility’. We use professional responsibility to connote a moral or professional obligation to ensure that a particular task is adequately performed. Contrastingly, we use the term ‘legal duty’ in relation to a legal duty of care in negligence, which is different in its focus and content. Such professional responsibilities and legal duties may coexist or may overlap [15]. However, it is important to avoid conflating professional responsibilities and legal duties, and to be clear about the distinction between the two.

As we will illustrate in the ‘Guiding legal and ethical aspects’ section below, current recontacting practices are insufficiently coherent and uniform to establish a legal duty to recontact. However, practice could change in the future and professional consensus about the importance of recontacting may lead to the establishment of a legal duty. The absence of clear legal duties to recontact leads, as a consequence, to an uncertainty in terms of who is legally responsible for what. In the absence of legal stipulations, codes of conduct and common practice are reviewed in cases of potential litigation. Therefore, we consider the term ‘responsibility’ more appropriate at present, and more responsive to future changes in ethical, normative, and legal approaches to recontacting. It is also more suitable to consider a ‘collectivised’ responsibility that can include patients, their relatives and HCPs, and potentially other stakeholders who may also play a role in updating HCPs about a reinterpretation of a variant of uncertain significance [9]. Thus, we will use the term ‘responsibility’ henceforth.

## Overview of current knowledge and evidence on recontacting

While there is still limited empirical evidence describing issues surrounding recontacting, the few studies conducted in Europe provide important information. Below we present data regarding views and experiences on recontacting from different stakeholders.

### Systematic review

We have found only one peer reviewed systematic review of the literature on recontacting in clinical genetics [6]. This review highlights the trend to consider recontacting as ethically desirable from the perspective of efforts to promote effective healthcare for all, although it may be neither fulfilled in practice nor logistically feasible. This trend still continues in more recent literature [15]. The main barriers identified by the review are lack of HCP time and resources, and the most common proposals to overcome these practical barriers revolve around the idea of HCPs, patients and laboratories sharing some responsibility for recontacting [6]. Furthermore, the authors note that ethical/legal/psychosocial reasons for and against recontacting vary in the literature. For example, new genomic information is seen as having significant implications for patients and family members concerning their health, reproductive decisions, lifestyle choices, employment, and psychosocial wellbeing. However, recontacting may also affect patients negatively, potentially causing anxiety and concerns over health and economic activity, and being perceived as an intrusion into privacy [6]. Accordingly, the review brings into focus that recontacting is consistently seen as more compelling for relevant and clinically valid information than for information that is less certain or less practically useful. The authors observe that a general responsibility to recontact in clinical genetics is currently not applicable and conclude that a useful way to advance the debate is to clarify *in what situations* recontacting can be regarded as an appropriate standard of care rather than considering a more general responsibility to recontact [6]. While this is reasonable, it remains to be determined what constitutes the key factors defining any one ‘situation’, as well as, from the procedural perspective, how to facilitate recontacting in the face of professional and operational constraints. The term ‘appropriate standard of care’ may also imply legal responsibilities as well as ethical duties.

### Patients’ views

The published literature describing patients’ views on recontacting is limited.

Carrieri et al. [14] analyzed the views of patients affected by various genetic conditions in the UK. Those interviewed tended to consider recontacting as desirable and, as previously reported in patient-centered research about the general attributes of genetic services [21], a sign of good quality care. However, patients appeared to have mixed expectations and views about the ‘significance’ or type of new information they would like to receive [14]. This is in line with literature on how patients perceive the clinical validity and personal utility of genetic testing in the clinical, research and commercial arenas [22, 23]. An important finding from the UK study [14] was the potentially complex psychosocial impact on patients and family members of receiving new information, irrespective of whether this information is linked to an improvement of health outcomes. Even patients who had been recontacted and had found it to be beneficial admitted that the actual *experience* of the recontact triggered complex psychological responses. Patients recognized the need to coordinate recontacting with timely access to clinical services, so that they can be offered the opportunity to discuss the new information and its implications with HCPs [14]. These considerations contribute to research that highlights the potentially negative psychosocial consequences of being recontacted [24, 25]. Moreover these considerations corroborate research that showed how patients are supportive of results being given by qualified HCPs and, in particular, by genetic counsellors [26].

A questionnaire study from the Netherlands [27] examined perspectives on recontacting of parents of children affected by intellectual disability. All these children were previously examined by karyotyping and sequencing of specific single genes without a specific diagnosis being reached. As the availability of high-resolution SNP array and trio exome sequencing made re-evaluation possible, parents were contacted either by telephone or letter. Most interviewed parents had positive opinions on recontacting, though it might have evoked emotional responses requiring deeper enquiry. Interestingly, about 36% of the patients contacted by telephone made a new appointment for re-evaluation, compared with only 4% of those approached by letter. The telephone call gave parents the opportunity to make an appointment directly, and also to ask many questions. Of note, almost 90% of the parents deemed the patient should not be responsible for initiating recontacting and mainly identified the clinical geneticist as the HCP who should take responsibility [27].

Also in the Netherlands, Halbersma and colleagues (personal communication) investigated patient perceptions of current recontacting practices by semi-structured in-depth interviews. The specific circumstance was a variant reclassification in over 150 families investigated for a hereditary cause of cardiomyopathy. Most reclassifications involved a variant currently considered to be less likely to be pathogenic, in other words a reclassification from likely pathogenic to VUS. At the time over 40 patients had been informed by letter explaining the variant reclassification and its consequences, and inviting them to contact the genetics department if they had questions, which surprisingly none of them did. Subsequent in-depth interviews with ten of these patients showed that they had appreciated being recontacted about the reclassification and felt that they needed no additional information. However, it was apparent that their understanding of the information was sometimes inaccurate (e.g. incorrect conclusions about transmissability and implications for their relatives).

A recent American questionnaire study [28] examined the feasibility, efficacy and patient opinions on recontacting by letter in a setting of adults previously affected by endocrine tumours (medullary thyroid carcinoma, phaeochromocytoma, paraganglioma). These patients were likely affected by a hereditary syndrome, but genetic testing at the time of initial assessment could not be completed, if performed at all. Almost all the patients who completed the survey deemed it appropriate for them to be sent information on new developments which could have implications for their or their family’s health, even though a minority were distressed by this. Despite the alleged positive reception, only a third further discussed the possibility of genetic testing with their doctor, and less than 10% actually underwent testing. The authors consequently questioned the effectiveness of this approach and proposed that research be pursued on more effective methods with improved patient satisfaction.

Overall, the studies currently available demonstrate that patients value being recontacted by HCPs, although this can represent for them a psychologically complex and stressful experience and may not lead to (medically) appropriate actions.

### Views from HCPs and clinical genetics laboratory professionals

One survey study administered to 105 genetics centres in Europe [13] (including 20 UK centres [11]) suggests that most centres in Europe have recontacted patients in the light of new significant information, but only a minority reported recontacting as a *routine* activity [13]. The survey answers revealed a multiplicity of understandings of the term ‘recontacting’; some respondents appeared to consider routine follow-up programmes or even the post-test result counselling to be part of recontacting [13]. This observation was mirrored in empirical research with patients and HCPs conducted in the UK [14].

The most common barriers for recontacting mentioned by European HCPs included lack of resources (time, reimbursement, staff, infrastructure), concerns about potential negative psychological consequences of recontacting for the patients, existing policies or laws that prevent HCPs from recontacting, and difficulties in locating patients after their last contact. Variation between European genetics centres in terms of procedures related to recontacting may be due to the highly variable evolution of the disciplines of medical genetics and genetic counselling across different European countries [13].

In line with the European survey results, Carrieri et al. [11] reported that recontacting occurs in the UK mostly in light of new information that HCPs consider to be clinically relevant for the patient. However, there are no standardized practices. HCPs attributed the ad hoc nature of current recontacting practices to a lack of resources (time, staffing and infrastructure). This means that there is no systematic way to ensure that all patients seen in a clinic who may benefit from new information are identified and recontacted [11]. This links recontacting to the broader issue of the equitability of current healthcare service provision [15]. Professionals’ and patients’ associations can also substantially contribute in updating HCPs and patients with new relevant information on specific conditions and help facilitate the recontact.

In the Dutch study on recontacting of parents of children affected by intellectual disability the researchers found recontacting feasible but very time-consuming, and a massive additional responsibility for clinical geneticists [27]. Overall, the genetic professionals deem recontacting patients over time valuable for the patient, but burdensome and very difficult to systematically realize in practice, even impossible in some scenarios due to legal and infrastructure barriers.

### Professional responsibility for recontacting

The EuroGentest (EG) and the ESHG Guidelines for diagnostic next-generation sequencing, published in 2015 [10], state that Genetic laboratories are ‘not expected to re-analyze old data systematically and report novel findings’, however, ‘if at a particular moment, it is decided—by the lab or by the community of experts in the disease—to change a variant from one class to another, the laboratory is responsible for reanalyzing the available data, re-issuing a report on the basis of the novel evidence, and also re-contacting referring clinicians for the patients that are possibly affected by the new status of the variant’ [10] (p. 5). Although some responsibility for recontacting is acknowledged in principle to fall to the genetic laboratories, the practicability remains elusive.

Vears et al. [29] propose points to consider to laboratories offering diagnostic genome analysis. In line with the EG-ESHG guidelines [10], they also support the view that laboratories do not have an obligation to routinely reanalyse data but rather in response to a specific request initiated by the patient or their clinician. However, if the information on reclassification of a given variant becomes available to the laboratory, then it would be considered good clinical practice for those laboratories to identify patients carrying that variant from their database and issue a new report to the original referring clinician [29]. It is also worth noting that, in the wider context, laboratories might have different rules in reporting, such that at present there is inconsistency, not only concerning whether or not a VUS is reported, but also sometimes on variant classification, even using the same guidelines for interpretation.

El Mecky, Johansson et al. (personal communication) undertook an online focus group of all clinical genetics laboratories in the Netherlands to discuss their current practice and views about future practice regarding the reclassification of variants, as well as which reclassifications are communicated from laboratory to clinician. Preliminary results indicate differing views on which reclassifications merit recontacting from laboratory to clinician. Furthermore, no systematic approaches exist for laboratories to actively reinterpret variants (i.e. periodically reviewing new evidence on genetic variants in the laboratory’s database, unprompted by patient or clinician), but some participants desired to implement such procedures.

The UK study [11, 15] reports a lack of agreement between genetic HCPs and clinical scientists in the laboratory about whose role it should be to monitor the changing interpretations of variants so that appropriate recontacting can be triggered.

The lack of a professional consensus was cited by respondents as another important cause of the ad hoc nature of recontacting. Some HCPs expressed the concern that they could be legally liable either if they recontact patients or if they fail to do so [15]. Interestingly, Carrieri et al. [11] also found a potential tension between a patient’s right not to know and HCPs’ responsibility or duty to recontact [30, 31]. For example, many survey respondents answered that they would, in a hypothetical situation, recontact patients and family members in light of new clinically relevant information, even if the patients had indicated they would not want them to do so. This perspective is, to some extent, related to the ESHG recommendations on the use of whole-genome sequencing in healthcare, in particular that: “Patients’ claims to a right not to know do not automatically over-ride professional responsibilities when the patient’s own health or that of his or her close relatives is at stake” [3] (p. 583). Given the hypothetical nature of a previously expressed preference not to be recontacted, and the potential responsibility of HCPs to the patient’s relatives, HCPs may consider whether the relevance of the new information to be conveyed to the patient and/or their family would justify a re-exploration of the earlier decision of the patient, or even a disclosure in the case of serious and actionable health risks, particularly if not previously anticipated. Moreover, the background document on consent and confidentiality of the UK Joint Committee on Genomics in Medicine [32] suggests that consideration should be given to viewing consent as a process, rather than as a one-off event. This view could indicate that HCPs should consider the decisions made during the initial consent process to be dynamic over time.

A suggestion made by both patients and HCPs interviewed in the UK was that recontacting could be a ‘joint venture’ between patients and HCPs; that is, that patients and HCPs could share some responsibility for recontacting [16, 17]. This argument was mainly based on two factors: (i) the issue of the lack of resources to implement recontacting sustainably within resource-constrained healthcare service, and (ii) discourses about patients’ responsibility to manage their own health (see Section 'Guiding legal and ethical aspects'). However, placing an onus of responsibility on the patient or family has the potential to introduce inequity and to maintain or exacerbate disparities in health and health care. Those who are more burdened by disease personally or in their family, who are unemployed or living in poverty, who are less articulate or less educated, who have less internet competence or access, or who have any cognitive impairment, may be less likely to rise to the challenge of initiating recontact when it might be appropriate. This may result in HCPs addressing more demands of people with elevated social advantages but whose health needs may be less, which—as described in Tudor Hart’s Inverse Care Law—may reinforce pre-existing inequities [33]. It should be an aim to avoid this outcome.

## Guiding legal and ethical aspects

### Legal aspects

There are two main areas of law which govern recontacting in Europe: (i) negligence/duty of care, and (ii) data protection. In addition, the regulatory frameworks of individual nation states which govern medical practice and genetic testing may have relevance to aspects of recontacting. However, these will vary from state to state and a comprehensive review is outside the scope of this article.

#### Negligence

In Europe, the law of negligence differs across nations, and the common law approach (taken in England & Wales, and Ireland) is different to that in civil law jurisdictions.

##### Common law approach

HCPs owe a duty to take reasonable care of their patients. To succeed in a negligence action (tort), a person must show that there has been a breach of this duty of care which has caused harm [34, 35]. The standard of care is judged by reference to a responsible body of professional opinion, or in other words, the standard practice of the profession or specialty, as long as that practice could not be rejected as incapable of standing up to rational analysis (*Bolam v Friener Hospital Management Committee* [1957] 2 All ER 118; *Bolitho v Hackney Health Authority* [1998] AC 232; *Montgomery v Lanarkshire Health Authority* [2015] AC 1430). Currently, recontacting occurs in the clinical genetics context largely in an ad hoc manner. In the absence of consistent practice, or any professional consensus about a professional responsibility to recontact, a legal duty of care is unlikely to arise at present. However, the nature of common law is such that, should practice change in the future, there may be sufficient weight of practice and professional consensus about the importance of recontacting to establish a legal duty to recontact.

##### Civil law approach

According to the civil law and the tort law, three core facts should be demonstrated to prove the professionals’ duty: a *fault*, a *damage*, and a *causal link* between the two. The fault should consist, in health law, in having failed to provide a standard of care [36]. This standard of care is evaluated by the judge according to the medical knowledge at the date of the damage and can either consist of the commission or omission of an act. Among the faults that jurisprudence considers as impinging on patients’ rights and autonomy (such as information, consent) are faults which can be compensated. The damage should usually be proven by the patient and can include loss of an opportunity for intervention regarding his medical condition. The patient should also characterize the link between the fault and the damage. These rules are implemented differently in the different European countries, which can adopt specific provisions regarding medical liability and command discretion in interpreting the scope of the fault and the burden of proof.

#### Data protection

As with all areas of medical practice, data protection law regulates key aspects of the processing of information about patients. It provides a framework to allow patients to apply for access to their personal data. This will allow proactive patients to recontact their HCPs to seek access to their personal data (although this does not include a requirement for reanalysis or updating of their medical information). However, the data protection framework does not provide a means for patients to compel HCPs to recontact them without application on their part. Most aspects of HCPs recontacting patients are likely to be non-controversial in many countries, and permitted by data protection law, providing that there is appropriate provision of information and consent to the use of data. Article 9 of the General Data Protection Regulation [37] governs the processing of genetic and medical information, which is a special category of personal data. Such data may be processed where a person has given explicit consent or under the scientific research exemption or for substantial public interest. In the absence of such consent, including if a patient has refused consent to be recontacted, the HCP aiming to conclude a diagnosis for the patient and their family benefit, could rely on either Art 9(g) (substantial public interest) or (h) (medical diagnosis) to make their conduct compliant with data protection law; however, the result should be kept by the physician if the individual has claimed his right not to know.

### Ethical aspects

HCPs, as revealed through research studies and professional debates [15, 28], have highlighted an urgent need for guidance about the circumstances in which recontacting should be considered a good standard of care, and about lines of responsibility [13]. Such guidance would reduce the current lack of clarity that HCPs are experiencing in relation to the clinical and medico-legal aspects of recontacting. Ideally, it should also lead to a more consistent and equitable healthcare service provision around recontacting.

#### Beneficence, non-maleficence, autonomy and justice

With respect to how recontacting could benefit patients, new information conveyed when a patient is recontacted might entail clinical or personal utility [22, 38, 39], and potentially improve the health of the patient or family; for instance, through directing them to appropriate management strategies. However, it is not always certain that the overall outcome, including psychosocial aspects, will be significantly improved. In a study from the Netherlands, for instance [27], 17% of parents recontacted for their child’s intellectual disability mentioned positive emotions, such as hopeful and relief, and negative emotions, such as grief, anger, fear and frustration. Indeed, recontacting might represent a source of anxiety and stress for the patient and family members, as it might be perceived as an intrusion of privacy and breach of their right not to know. The new contact might elicit memories of an emotionally difficult episode of their life, which had meanwhile been ‘archived’ or put aside, and which they might not wish to re-experience. The new information provided might also have an impact on practical, financial and legal aspects of their situation, for example their eligibility for health and life insurance, adoption applications, and so on. The decision on whether or not to recontact a patient at a certain moment would require careful weighing of all the above-mentioned factors and, inevitably, with flexibility. Decisions will inevitably be made on a case-by-case basis.

On the other hand, and mirroring the ongoing debate around the issue of disclosure of unsolicited findings (UFs; findings beyond the initial clinical question) to participants/patients in clinical, research and commercial arenas [40–42]—for certain types of results (i.e. high penetrance, severity of phenotype)—there seems to be a progressive opening to consider the ‘Justified hard paternalism’ framework [43]. According to this framework the ‘duty to warn’ is interpreted as a ‘duty to rescue’, which is felt to be obligatory beneficence. Under this framework, the cardinal genetic counselling tenets of patient autonomy and the right-not-to-know (central in the ‘respect’ pillar of the clinical ethics framework) [44, 45] are currently being challenged. Consent procedures might also shift accordingly from an ‘opt in’ to an ‘opt out’ framework, as well as items discussing the option of data sharing, for instance with other family members, are shifting from ‘choice’ to ‘information’. In this context, it will be essential to carefully consider, in dialogue with all stakeholders, and with consideration of legal and ethical standards to protect patients’ autonomy and right not to know, whether a patient’s one-off choices about hypothetical future scenarios (including on recontacting) would be considered as strictly binding for all types of information.

In this respect it is worth mentioning a pilot project run by the Clinical Genetics working group of the Italian National Society of Human Genetics (SIGU) in 2009, which resulted in the document ‘Continuità Assistenziale in Genetica Clinica: il ricontatto’ (‘Continuity of care: recontacting’ [46], document in Italian only). The document suggested that, whatever the policy of the service on recontacting, it should have been clearly explained to the patient. In a case where active recontacting would have been a potential option, written consent should be obtained from the patient, in order to be compliant with privacy policies. A model consent form was also suggested. A subsequent survey found that, despite a general agreement regarding recontacting being in principle good practice, only a net minority of centres declared to have adopted a bespoke consent. The reasons provided included a written consent being unnecessary and fear of litigation in case recontacting would not have been executed (personal communication).

Regardless of the model of consent that is eventually adopted, in order for patients to be able to make an informed decision about testing it is important that a discussion about recontact takes place during the consent process. Indeed, a study by Ayuso et al. [47] proposed that recontact should be included as one of the core elements on consent forms provided to patients undergoing diagnostic genomic sequencing. Another, more recent study undertook a systematic search for consent forms available online and in English that were in use for high-throughput diagnostic next generation sequencing [48]. Fifty-eight consent forms were analysed to assess their policies for recontacting patients, and the mechanisms for doing so. Only 23/58 forms mentioned some form of recontact for clinical purposes. There was considerable variation regarding who would initiate recontact if new information was identified: forms most commonly stated that the laboratory would recontact the clinician (8 forms), or the patient (8 forms), or recommended that the patient should recontact the clinician to check if their results have changed (6 forms). Only in four cases patients were asked to give separate consent for clinical purposes. It is important that the roles and responsibilities of the laboratory, clinician, and patient are clearly defined to ensure all parties are aware of their part in the process. The consent process provides the best opportunity for such clarification as a specific conversation about recontacting can be initiated between clinician and patient when seeking to cover all points of information. From a legal perspective, discussing consent for future recontacting may be key to ensuring alignment with data protection requirements and protection of persons’ rights.

From the perspective of social justice the effort needed for recontacting has to be proportionate and sustainable. Though ideally all patients who may benefit from new information are identified and recontacted, there is no guarantee that each clinic would have the resources to meet these ideals, and HCPs are allowed to recontact so inequity might result.

## Discussion

In clinical genetics, recontacting patients over time is sometimes performed in Europe, with HCPs reporting that it mostly occurs when new, clinically relevant information or new techniques become available [13]. However, there are no standardized criteria, practices or systems in place for recontacting to occur [13]. Importantly, there is a lack of economic evidence about the relative costs and consequences of strategies to recontact patients, either in general or for individuals with specific diseases. Recontacting thus raises the broader issue of equity in current healthcare service provision, in that there is no systematic way to ensure that all patients who may benefit from new information are identified and recontacted, and also that finite healthcare resources may force decision makers to prioritize elsewhere. Even if a systematic approach was proposed, there is no guarantee that each clinic would have the resources to meet these ideals, so inequity would result.

The few existing empirical studies [11, 13, 15, 27, 28] found that HCPs deem recontact theoretically possible, but there is significant concern among HCPs that they may be held legally liable whether they do, or do not, recontact patients [11, 15]. Both HCPs and patients express concerns about a lack of clarity over roles and responsibilities [14, 15]. This lack of clarity is further complicated by the fact that genetic and genomic testing is increasingly offered by specialties that do not necessarily have an ongoing relationship with a patient [49, 50], and non-genetic specialists may not be equipped to communicate information on reclassified variants. HCPs see the need for professional guidance about whether, and in what circumstances, to recontact, and whose responsibility this should be [13, 15].

Furthermore, both HCPs and patients have expressed concerns about the feasibility of routinely recontacting within the current resource constraints of healthcare systems [13, 15, 17, 27]. An ‘opportunity cost’ argument has been developed in favour of timely recontacting being an effective use of healthcare resources, considering the potential preventative value of correct information, its psychosocial benefits and ultimately improving health outcomes. Moreover, when dealing with inherited conditions, the ‘cost-effectiveness’ analyses should also include the repercussions on family members, and not only the patient in isolation. To be convincing, however, this premise requires the support of evidence from the field of health economics. An additional consideration that favours recontacting, from a clinical perspective, is that its implementation may provide opportunities to gather more information about the natural history of genetic conditions and their impact on patients and families over time, contributing to interpretative knowledge.

In summary, the evidence currently available [13, 14] suggests that recontacting patients is viewed as desirable by the relevant stakeholders, notwithstanding its complexities and objective hurdles. At present, there is no economic evidence to support or to oppose to its introduction.

The development of professional guidelines and appropriate tools and procedures to achieve this implementation would be welcomed.

A precondition to making recontacting feasible is the simplification of the process as much as possible, and this can only be achieved with suitable informatics tools, appropriately set up.

Electronic medical records, genetics laboratory databases, and patients’ registries, all represent powerful tools to identify, monitor and reach patients who might benefit.

The genetics laboratories are the first repositories of the variants which might be reinterpreted over time, and as such the ideal point of departure for triggering recontact through the referring clinicians. Centralization of laboratory facilities and their collaboration with data sharing tools and curated databases might simplify this process. Professionals’ and patients’ associations can also substantially contribute in updating HCPs and patients with new, relevant information on specific conditions, and facilitating the recontact.

While identifying different stakeholders in sharing responsibility to recontact, including patients, genetic and non-genetic HCPs, and lab specialists, such shared responsibility should be seen as a pragmatic way forward until best practices have been identified. Meanwhile we should be aware of the risk that expecting others to assume responsibility may result in failure to take any action.

If a routine follow-up of the patient is in place with another specialty, their involvement should be considered, as that specialist will have insight into the present situation of the patient and the process may be more streamlined.

As genetic and genomic testing becomes progressively more integrated in mainstream medicine, the identification of the HCP who should take the initiative in re-evaluating the case of the patient and/or recontacting them may become even more intricate. The implementation of multidisciplinary teams (MDTs) involved in the care of rare and/or complex cases has been introduced in some settings and can support good practice. However, this may require a long time to be consistently and systematically implemented and might not apply to all possible situations.

Whether or not MDT meetings take place, it is in any case desirable for collaboration and coordination to be established among all the HCPs involved in the care of a patient. This would create opportunities to evaluate individual cases in a collegial way, assist in the integration of the resources and information available, and arrive at the most reasonable decision on how to manage individual cases, including the practical delivery of care.

Considering recontact practices over time may offer an opportunity for HCPs, policy makers and other stakeholders to focus on long-term strategies (as opposed to the prevalent ‘short-termism’) for the delivery of an efficient and sustainable health service, which would proactively contribute in sustaining the positive circle of the implementation of e-health records.

An open dialogue will be important to promote public engagement, enhance recognition of the need for data sharing, and build confidence in the mechanisms for enabling that process. If such demonstrably trustworthy systems can be built, this will be to the great benefit of patients and their families.

## Recommendations

### 1. If possible, recontact should take place for findings with clinical or established personal utility, even though currently there is no duty to do so

In clinical genetics, recontacting for updating patients with new, meaningful information related to their diagnosis or their previous genetic testing may be justifiable and, where possible, it is desirable. The consensus about the type of information to disclose that should trigger the recontacting process converges around clinical and personal utility.

### 2. The decision to recontact should be based on the best interests of the patient/family

The decision to recontact should be guided by the best interests of the patient/family and on the basis of previous agreements with the entitled person(s). The anticipated benefits of recontacting should outweigh the risks to the patient/family. Any tensions between the best interest or wishes of a patient versus those of the family should be carefully considered. It will be important to consider whether the new information for professionals to convey is linked to the original clinical question or not, and to take account of the previous, hypothetical choices of the patient regarding being informed of possible collateral or unsolicited findings.

### 3. Recontacting should be sustainable for the health care system and its workforce

The effort needed for recontacting has to be proportionate and sustainable, from the perspective of social justice. The benefits for patients and their families should be balanced with the impact that the recontacting activities may have on HCPs’ work and the service as a whole in order to ensure the just offer of clinical care. This should not prevent HCPs and services from offering excellent, equitable, trusted clinical care in the future.

### 4. The allocation of dedicated resources for activities on recontacting should be considered

Sustainable and equitable recontacting cannot be achieved without suitable informatics tools, appropriately set up. Ideally these should be integrated into the Electronic Clinical Records and the databases of diagnostic genetics laboratories. Implementation of patients’ registries also represents a powerful tool. Resources should be allocated for research on recontacting procedures. Research is needed to further understand recontacting activities and how they could best be organised, including research on HCPs’ activities and development of informatics tools.

### 5. Recontacting should strive to be equitable for patients from all socioeconomic backgrounds and contexts

When implementing recontact in practice, it will be important to define strategies to ensure we are addressing the patients’ needs on the basis of clinical priority while tackling inequalities in healthcare access and individual resources. People who are more burdened by disease or poverty, less articulate or educated, cognitively impaired, with less IT access or fewer skills, merit special consideration. Particular attention should be devoted to patients whose test was conducted while they were children, whose legal status might have changed, and who might not have been fully informed about their previous contacts with a clinical genetics service and their outcomes.

### 6. There is a need for professional consensus about what constitutes good practice regarding consent for recontacting

It will be important that HCPs who are offering genetic testing/counselling address the practical and ethical issues of recontacting. HCPs should clearly state what may happen, assess and record each patient’s views, and agree on the way forward, involving patients when possible. A wider debate on the evolution of consent and confidentiality issues in clinical genetics will be needed.

### 7. Recontacting should be a shared responsibility with patients

Where patients relocate or change their mind in respect of being recontacted, it is important for the genetics service to be kept informed. Placing additional responsibility on patients to initiate recontacting might be seen as a pragmatic solution to the current lack of resources and infrastructure but risks distracting services from their most important tasks and reinforcing inequities in health care. Any patient-led recontacting requires an effective partnership between patient and genetic services, in which the patient is offered education on why, how and when s(he) might consider establishing a new contact with a genetics centre.

### 8. Recontacting should be a shared responsibility with the genetic laboratories

Genetics laboratories are the natural repositories of the genetic variants identified in patients, which might be reclassified over time. As such, they might represent the ideal point of departure for triggering the recontacting process, by alerting the referring clinician about the new information. Laboratory scientists might therefore share responsibilities in recontacting. Multi-specialty collaborations between HCPs and clinical genetics laboratories appear to be one of the most effective ways to implement recontacting in clinical practice in a smooth and equitable way. In case the recontacting might involve a direct contact between the laboratory and the patient, where this is allowed or required by the local healthcare system, appropriate information on the patient’s recorded consent on the matter should be obtained.

### 9. Data sharing should be promoted

The centralization of laboratory facilities, progressive adoption of data sharing tools and of curated databases might support and simplify both the re-interpretation of VUSs and the implementation of the recontacting process. An open dialogue to promote patient and public engagement and enhance recognition of the importance of data sharing, explaining the clinical utility of whole exome/genome sequencing approaches, as well as their complexities, will be instrumental in developing these systems for the benefit of the patients and their families.

### 10. Other stakeholders should also share responsibility for recontacting

Non-genetic HCPs, Professional Networks (as for instance European Reference Networks for Rare Disorders) and Patients’ Associations, represent crucial stakeholders who should also be involved in the recontacting debate. Networks of professionals and patients can also contribute to, and facilitate, the recontacting process by updating both HCPs and patients with new relevant information.

In health care, if routine follow-up of a patient is in place with non-genetic specialists, their involvement should not only be considered but possibly represent the main route for conveying new or updated information to the patient.

### 11. Do the best you can with limited resources

At present, all healthcare systems throughout the EU are working with fixed and limited budgets and often struggling to maintain even basic services. In this scenario, and without the appropriate resources, it is difficult to expect Genetics Services to recontact patients in a consistent and systematic way, if at all. We propose that this activity will be included within the workload of Genetics HCPs but for that it must be appropriately resourced. There is a need for economic evidence to inform decisions about which strategies enable the most cost-effective use of healthcare resources to recontact patients.

### 12. Each country should define its own organizational policy on the recontact process

As European countries have different healthcare systems and legal frameworks it will be essential to identify in each nation the relevant stakeholders, the protocol options, and the resources to be allocated before any systematic recontacting can be operational. While this process might require a considerable amount of time, we urge that a dialogue be started among stakeholders at the level of each country to promote a harmonisation of the procedures at the national level, even if it cannot yet be achieved at the European level. Regional and national experiences can inform European initiatives for harmonisation. Resources should be allocated for pilot programmes implementing recontacting procedures.

### 13. More research is needed to inform responsible recontacting processes

Additional research on how stakeholders, including patients and families, experience recontact, and how it impacts the economics of healthcare systems, should be supported in order to help inform the development of a responsible re-contacting process and develop tools to support dynamic consent procedures.

## Closure

While genetic services seem to be the central focus now, genomic testing is becoming increasingly integrated within mainstream medicine, which will contribute to the development of a new ‘genomic medicine’ vision, even if it may not come to pass in practice exactly as envisaged. In addition, experience from evolving frameworks and processes, such as screening programmes and the return of results from research using biobanks, will be informative. We expect to revise this document in the light of such ongoing experiences in the coming years.

## References

[1] Dondorp WJ, de Wert GM (2013). The ‘thousand-dollar genome’: an ethical exploration. Eur J Hum Genet.

[2] Richards S, Aziz N, Bale S (2015). Standards and guidelines for the interpretation of sequence variants: a joint consensus recommendation of the American College of Medical Genetics and Genomics and the Association for Molecular Pathology. Genet Med.

[3] Van ElCG, Cornel MC, Borry P (2013). Whole-genome sequencing in health care. Eur J Hum Genet.

[4] Howard HC, Iwarsson E (2018). Mapping uncertainty in genomics. J Risk Res.

[5] Lucassen A, Houlston RS (2014). The challenges of genome analysis in the health care setting. Genes.

[6] Otten E, Plantinga M, Birnie E (2015). Is there a duty to recontact in light of new genetic technologies? A systematic review of the literature. Genet Med.

[7] Burton, H, Genetics and mainstream medicine: service development and integration. 2011, PHG Foundation.

[8] Hirschhorn K, Fleisher LD, Godmilow L, Howell RR, Lebel RR, McCabe ERB (1999). Duty to Re-contact. Policy Statement: Social Ethical and Legal Issues Committee of the American College of Medical Geneticists. Genet Med.

[9] Shirts BH, Parker LS (2008). Changing interpretations, stable genes: responsibilities of patients, professionals, and policy makers in the clinical interpretation of complex genetic information. Genet Med.

[10] Matthijs G, Souche E, Alders M (2015). Guidelines for diagnostic next-generation sequencing. Eur J Hum Genet.

[11] Carrieri D, Lucassen AM, Clarke AJ (2016). Recontact in clinical practice: a survey of clinical genetics services in the United Kingdom. Genet Med.

[12] Rantanen E, Hietala M, Kristoffersson U, Nippert I (2008). Regulations and Practices of Genetic Counselling in 38 European Countries: the Perspective of National Representative. Eur J Hum Genet.

[13] Sirchia, F, Carrieri, D, Dheensa, S, Benjamin C, Kayserili H, Cordier C, et al. Recontacting or not recontacting? A survey of current practices in clinical genetics centres in Europe. Eur J Hum Genet. 2018;26:946–954. 10.1038/s41431-018-0131-5 PMC6018700 29681620

[14] Carrieri D, Dheensa S, Doheny S (2017). Recontacting in clinical practice: the views and expectations of patients in the United Kingdom. Eur J Hum Genet.

[15] Carrieri D, Dheensa S, Doheny S (2017). Recontacting in clinical practice: an investigation of the views of healthcare professionals and clinical scientists in the United Kingdom. Eur J Hum Genet.

[16] Carrieri D, Dheensa S, Doheny S (2017). Recontacting in clinical genetics and genomic medicine? We need to talk about it. Eur J Hum Genet.

[17] Dheensa S, Carrieri D, Kelly S (2017). A’joint venture’model of recontacting in clinical genomics: challenges for responsible implementation. Eur J Med Genet.

[18] Letendre M, Godard B (2004). Expanding the physician’s duty of care: a duty to recontact*?*. Med Law.

[19] Knoppers BM (2001). *Duty to Recontact: a Legal Harbinger?*. Am J Med Genet.

[20] Pelias MZ (1991). Duty to Disclose in Medical Genetics: a Legal Perspective. Am J Med Genet.

[21] McAllister M, Payne K, MacLeod R, Nicholls S (2008). What process attributes of clinical genetics services could maximise patient benefits*?*. Eur J Hum Genet.

[22] Bunnik EM, Janssens ACJ, Schermer MH (2015). Personal utility in genomic testing: is there such a thing?. J Med Ethics.

[23] Foster C, Watson M, Moynihan C (2002). Genetic testing for breast and ovarian cancer: cancer burden and responsability. J Health Psychol.

[24] Sharpe NF (1999). The duty to recontact: benefit and harm. Am J Hum Genet.

[25] Pyeritz RE (2011). The coming explosion in genetic testing - is there a duty to recontact?. N Engl J Med.

[26] Patient Charter, Genome Sequencing – What do patients think? 2015, Genetic Alliance UK: https://www.geneticalliance.org.uk/media/1924/patient-charter-genome-sequencing-what-do-patients-think.pdf.

[27] Beunders G, Dekker M, Haver O, Meijers-Heijboer HJ, Henneman L (2018). Recontacting in light of new genetic diagnostic techniques for patients with intellectual disability: Feasibility and parental perspectives. Eur J Med Genet.

[28] Romero Arenas MA, Rich TA, Hyde SM (2018). Recontacting Patients with Updated Genetic Testing Recommendations for Medullary Thyroid Carcinoma and Pheochromocytoma or Paraganglioma. Ann Surg Oncol.

[29] Vears DF, Sénécal K, Clarke AJ (2018). Points to consider for laboratories reporting results from diagnostic genomic sequencing. Eur J Hum Genet.

[30] Laurie G. Privacy and the right not to know: a plea for conceptual clarity. In: Chadwick R, Levitt M, Shickle D (eds). *The Right to Know and the Right Not to Know: Genetic Privacy and Responsibility.* Cambridge University Press: Cambridge, UK, 2014: p. 38.

[31] Hunter AGW, Sharpe NF, Mullen M, Meschino WS. Ethical, Legal, and Practical Concerns about Recontacting Patients to Inform them of New Information: the Case in Medical Genetics. In: Sharpe NF, Carter RF (eds). *Genetic Testing. Care, Consent and Liability*, John Wiley & Sons, Inc., 2006. 11746004

[32] Lucassen A, Hall A (2012). Consent and confidentiality in clinical genetic practice: guidance on genetic testing and sharing genetic information. Clin Med.

[33] Hart JT (1971). The inverse care law. Lancet.

[34] Laurie G, Harmon S, Porter G. *Mason and McCall Smith’s law and medical ethics*. Oxford University Press, 2016.

[35] Mitchell C, Ploem C, Chico V (2017). Exploring the potential duty of care in clinical genomics under UK law. Med Law Int.

[36] Koch BA, Bagińska E. Medical liability in Europe: a comparison of selected jurisdictions. 29. 2011: Walter de Gruyter.

[37] Regulation (EU) 2016/679 of the European Parliament and of the Council of 27 April 2016 on the protection of natural persons with regard to the processing of personal data and on the free movement of such data, and repealing Directive 95/46/EC (General Data Protection Regulation).

[38] Lesko L, Zineh I, Huang SM (2010). What is clinical utility and why should we care?. Clin Pharmacol Ther.

[39] Foster MW, Mulvihill JJ, Sharp RR (2009). Evaluating the utility of personal genomic information. Genet Med.

[40] Shkedi-Rafid S, Dheensa S, Crawford G, Fenwick A, Lucassen A (2014). Defining and managing incidental findings in genetic and genomic practice. J Med Genet.

[41] Souzeau E, Burdon KP, Mackey DA (2016). Ethical considerations for the return of incidental findings in ophthalmic genomic research. Transl Vision, Sci Technol.

[42] Middleton A, Morley KI, Bragin E (2016). Attitudes of nearly 7000 health professionals, genomic researchers and publics toward the return of incidental results from sequencing research. Eur J Hum Genet.

[43] Beauchamp TL, Childress JF. *Principles of biomedical ethics,* 7th edn. Oxford University Press: USA, 2013.

[44] Resta R, Biesecker BB, Bennett RL (2006). A new definition of genetic counselling: National Society of Genetic Counselors’ Task Force report. J Genet Couns.

[45] McCarthy Veach P, LeRoy B, Bartels D (2007). Coming full circle: a reciprocal-engagement model of genetic counseling practice. J Genet Couns.

[46] Belli S, Bedeschi F, Chessa L, Natacci F, Tenconi R, Zollino M, et al. ‘Continuity of care: recontacting’, 2009. https://urlsand.esvalabs.com/?u=https%3A%2F%2Fwww.sigu.net%2Fgdl%2Fmore%2Fsezione-id%2F5%2Farea%2F303%2FDocumenti%3Fpage%3D1&e=f6339128&h=d43e4005&f=n&p=y (Note: only SIGU members can access the document, password protected)

[47] Ayuso C, Millán JM, Mancheno M, Dal-Ré R (2013). Informed consent for whole-genome sequencing studies in the clinical setting. Proposed recommendations on essential content and process. Eur J Hum Genet.

[48] Danya F. Vears, Emilia Niemiec, Heidi Carmen Howard & Pascal Borry Analysis of VUS reporting, variant reinterpretation and recontact policies in clinical genomic sequencing consent forms Eur J Hum Genet. 2018; Published online 24 August 2018 10.1038/s41431-018-0239-7https://www.ncbi.nlm.nih.gov/pubmed/30143804 10.1038/s41431-018-0239-7 PMC6244391 30143804

[49] Burton H, Cole T, Fardon P. Genomics in Medicine. Delivering Genomics Through Clinical Practice, in Report of the Joint Committee on Medical Genetics. 2012.

[50] Burton H, Alberg C, Stewart A (2010). Mainstreaming Genetics: A Comparative Review of ClinicalServices for Inherited Cardiovascular Conditions in the UK. Public Health Genom.

